# Challenges and strategies to improve the availability and geographic accessibility of physicians in Portugal

**DOI:** 10.1186/s12960-017-0194-3

**Published:** 2017-03-23

**Authors:** Ana Paula Cavalcante de Oliveira, Gilles Dussault, Isabel Craveiro

**Affiliations:** Global Health and Tropical Medicine, Instituto de Higiene e Medicina Tropical, Universidade Nova de Lisboa, Rua da Junqueira, 100, 1349-008 Lisbon, Portugal

**Keywords:** Health labour market, Shortage of physicians, Geographical imbalances, Level of care maldistribution, Health workforce interventions

## Abstract

**Background:**

Shortages of physicians in remote, rural and other underserved areas and lack of general practitioners limit access to health services. The aims of this article are to identify the challenges faced by policy and decision-makers in Portugal to guarantee the availability and geographic accessibility to physicians in the National Health Service and to describe and analyse their causes, the strategies to tackle them and their results. We also raise the issue of whether research evidence was used or not in the process of policy development.

**Methods:**

We analysed policy and technical documents, peer-reviewed papers and newspaper articles from 1995 to 2015 through a structured search of government websites, Portuguese online newspapers and PubMed and Virtual Health Library (*Biblioteca Virtual em Saúde* (BVS)) databases; key informants were consulted to validate and complement the documentary search.

**Results:**

The challenges faced by decision-makers to ensure access to physicians were identified as a forecasted shortage of physicians, geographical imbalances and maldistribution of physicians by level of care. To date, no human resources for health policy has been formulated, in spite of most documents reviewed stating that it is needed. On the other hand, various isolated and ad hoc strategies have been adopted, such as incentives to choose family health as a specialty or to work in an underserved region and recruitment of foreign physicians through bilateral agreements.

**Conclusions:**

Health workforce research in Portugal is scarce, and therefore, policy decisions regarding the availability and accessibility of physicians are not based on evidence. The policy interventions described in this paper should be evaluated, which would be a good starting point to inform health workforce policy development.

**Electronic supplementary material:**

The online version of this article (doi:10.1186/s12960-017-0194-3) contains supplementary material, which is available to authorized users.

## Background

Imbalances in the geographical distribution of qualified Human Resources for Health (HRH) in rural or poor areas are observed in almost all countries in the world [1–7], including Portugal in spite of its small dimension [8–26]. Their impact is that access to health services is limited for segments of the population whose health needs may not be addressed adequately in a timely manner. The availability and accessibility of qualified health workers determines which services, and in which quantity, will be available to a population [1, 2, 5, 27]. In the context of the commitment of the member states of the United Nations Assembly to universal health coverage and to achieving the Sustainable Development Goals, this problem is a major challenge for policy-makers. Research on health workforce topics have been developed in the last 10 years almost exponentially, and evidence on what works and what does not work to respond to this challenge is available. A relevant research question is whether and how research results inform policy-making.

The aims of this article are to identify the challenges faced by policy and decision-makers in Portugal at the national level to guarantee the availability and geographic accessibility of physicians in the National Health Service (NHS) and to analyse their causes, the strategies to tackle them and their results. We also raise the issue of whether research evidence was (not) used in the process of policy development.

We first describe the data collection and analysis process and follow by describing how the three challenges of shortage, geographical and level of care imbalances were identified and analysed. A presentation of the various interventions developed by policy-makers to address these challenges follows. We then discuss the factors that influenced policy-making in relation to health workforce issues.

## Methods

This work is part of a holistic multiple-case study that includes three phases of data collecting. It aims to understand the process by which HRH policies addressing the geographical distribution of physicians are (not) informed by scientific evidence. This article reports the first and second phase of the case study, in which we analyse policy and technical documents, peer-reviewed articles and newspaper articles with the purpose of understanding the policy-making process in relation to the health labour market in Portugal. We conducted a structured search in PubMed and the Virtual Health ​Library (*Biblioteca Virtual em Saúde* (BVS)), in government and other relevant websites and in newspapers available online. In parallel, we consulted six informants (a researcher, three professionals from the Ministry of Health (MoH) and two from the NHS) to validate our data collection strategy and eventually give us access to unpublished documents. The search strategy is described in Additional file 1: Table S1. Selected documents were classified as (I) official documents of the Portuguese government, such as National Health Plans, relevant legislation, technical and policy analysis reports and policy statements; (II) scientific research documents including peer-reviewed articles and research reports; and (III) newspaper articles.

**Fig. 1:**
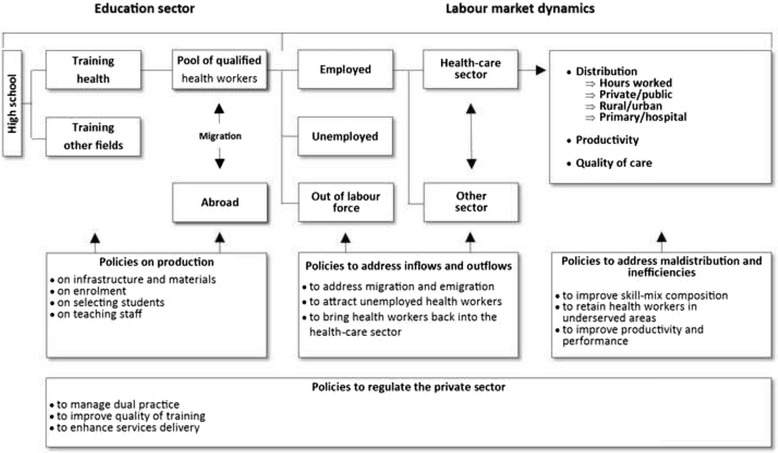
Health labour market and policy lever framework. Source: Adapted from Sousa et al. [28]

We used an adaptation of the health labour market and policy lever framework proposed by Sousa et al. (Fig. 1) [28], to improve the understanding of the dynamics of the health labour market and to identify relevant policy options [29]. The quantity of health workers that employers are willing to hire and the demand for health services by individuals, provider organizations or health planners define the demand for health workers [30, 31]. The number of health workers required to produce a certain set of health care services depends on a number of factors such as their competencies, available technology and productivity [29]. The number of health workers employed or willing to work in the health care sector constitutes the supply. It varies principally according to inflows from the education pipeline and from immigration, and according to outflows through emigration, career breaks, retirement and other forms of attrition [31]. Policy interventions can cover four areas according to their purpose: production, inflows and outflows, the regulation of the private sector and imbalances and inefficiencies in the distribution and utilization of health workers [28].

Health policy is “the process by which a problem is conceptualized, solutions and alternatives are formulated, decisions are taken, policy instruments are selected and programmes and strategies are implemented” [32] in order to respond to a social problem. Policy defines what decision-makers intend to do, and strategies specify how they plan to do it.

Data extraction was made by sub-categories using codes to answer the following questions:

1. What are the challenges faced by decision-makers at the national level in Portugal to ensure access to physicians?
2. What are the causes/determinants of these challenges?
3. What were the strategies implemented to address these issues?
4. What were the results of these strategies?

The program MaxQDA 2012 was used to facilitate the categorization and Mendeley to organize references.

## Results

The BVS and PubMed search (April 2015) identified 909 and 930 documents, respectively, for a total of 1426 documents after the exclusion of duplicates. The application of the inclusion criteria reduced the list of selected documents for full reading to 47, and six studies identified by key informants and other sources were added; Additional file 2: Figure S2 shows the process of selection of peer-reviewed article search. The website search identified 49 documents, of which 16 are policy documents and 33 are technical reports. The search of online newspapers identified 1454 potentially relevant articles, and 94 were selected for analysis. The full list of 155 analysed documents is available on request.

Most the articles identified in this documentary search (nine out of 12 documents) report that the authors did not have access to external financial support or did not mention any.

### Geographical access to physicians in the Portuguese National Health Service

The challenges faced by decision-makers to ensure access to physicians at the national level were identified as (a) a forecasted shortage of physicians, (b) geographical imbalances and (c) maldistribution by level of care.

#### Shortage of physicians?

Although the number of physicians in Portugal per 1000 habitants has been above the European Union (EU) 27 countries’ average between 1995 and 2013 [33], a shortage of physicians has been forecasted, particularly of general practitioners/family physicians (GPs) and of public health physicians in three scientific research documents, three political documents and one policy analysis document [13, 14, 19, 21, 34–36]. This was attributed to the low numerus clausus policy limiting entry in medical schools between 1979 to 2000, combined with the planned retirement of large numbers of physicians in the coming years [14, 19, 21, 25, 37–39]. In 1979, the numerus clausus was 805; in 1985, it dropped to 272; and in 2001, it was increased to 945 [40].

No analysis other than forecasting the numbers of future graduates and future retirees has been found. There is no monitoring of outflows to the private sector, to other sectors other than health or to other countries; dual practice is not monitored either [12, 23, 39, 41, 42]. Emigration flows are estimated by proxy indicators such as cancellation or suspension of registration and requests of Certificates of Good Standing [43, 44], which rose from 191 in 2009 to 650 requests in 2012 only in the South Regional Session of the Portuguese Medical Council [43]. In 2014, the total number of requests was approximately 1100 for the whole country [45], and between January and May 2016, there were another 226 requests [46].

#### Geographical imbalances

**Fig. 2:**
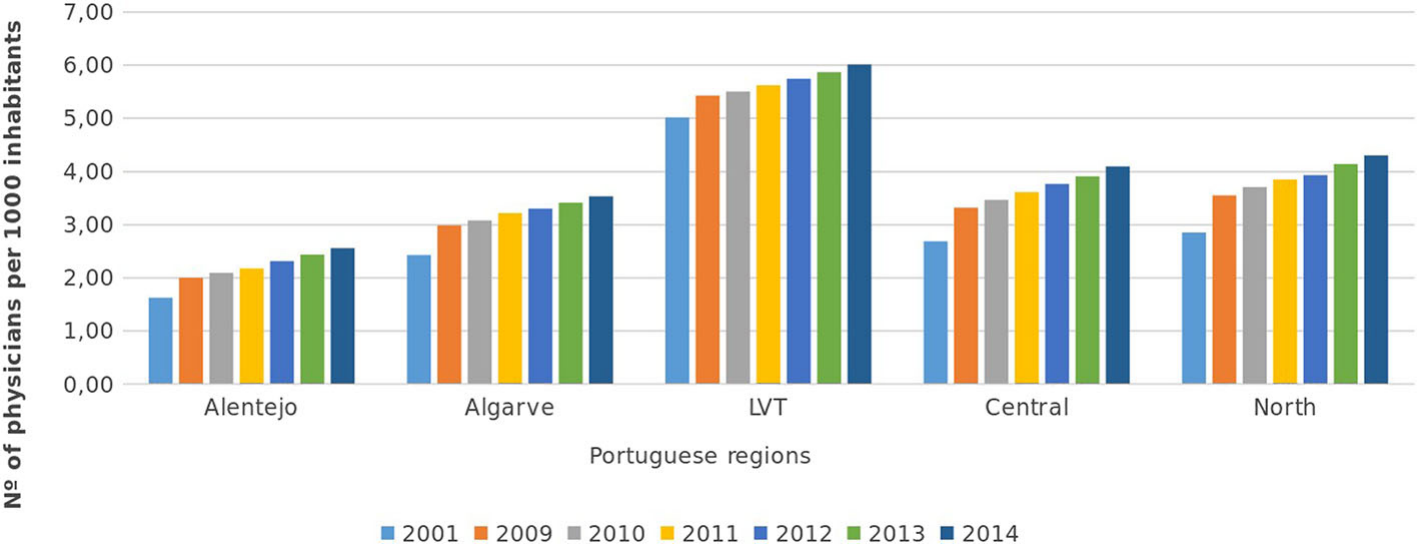
Number of physicians per 1000 inhabitants from 2009 to 2014 per region. Source: Fundação Francisco Manuel dos Santos [47]

The geographical maldistribution of physicians is acknowledged in Portugal; it is discussed in six scientific research documents, four political documents and nine policy analysis documents [8–26]. The distribution of physicians favours the three main urban areas of Oporto, Coimbra and Lisbon [8, 11, 22, 47, 48] (Fig. 2) where the most advanced technology and the oldest medical schools and teaching hospitals are found [10, 48]. In 2011, the Northern and the Lisbon/Vale do Tejo (LVT) regions, where 65% of the population resides, had 74% of NHS physicians; the Central region had 18%, Alentejo 4% and Algarve 4%, whereas they had 23, 7.5 and 4.5% of the population respectively [47, 49]. Portuguese private practitioners also tend to concentrate in the richer urban areas, as is the case in most Organisation for Economic Co-operation and Development (OECD) countries [50].

The maldistribution has been attributed to the lower supply of NHS beds and to the population’s lower purchasing power [8, 10] in disadvantaged regions. Another factor is the possibility of multiple employment in urban areas where the private sector offers additional remuneration opportunities [10].

#### Maldistribution by level of care

Another challenge identified in the documentation under review is the imbalanced distribution of physicians between primary care (PC) and hospital services; it is discussed in five scientific research documents and 11 policy analysis documents [11–16, 24, 34, 37, 42, 48, 51–55]; this is in spite of a stated policy to promote Family Health Care [11, 52, 54, 56], which includes the creation of Family Health Units [57, 58] consisting in multi-professional units which have organizational, technical and functional autonomy and that provide personalized health care to a given population [59, 60]. The first ones were implemented in 2007, and in late 2016, there are 459 covering 53% of the population.

The reason for the maldistribution was attributed to the low prestige of PC, to the lack of planning and to the limited number of internship places in family medicine as training capacity had not been developed in health centres [14, 34, 37, 52]. The problem is compounded by the ageing of the medical profession specially affecting GPs [42, 61–63], 75% of whom were above 50 years of age in 2011 [49], and by a wave of early retirements provoked by the austerity measures implemented after 2011 in the NHS [37, 49, 64].

### Strategies to address health workforce imbalances

A first attempt to define a strategy for the development of the health workforce was the publication, in 2001, of a “Strategic Plan to Education and Training in The Health Areas” [11, 19, 65]. It drew the attention to geographical and level of care imbalances and predicted a shortage of physicians in the coming years. In the context of political instability prevalent at that time, there was no follow-up [11].

The National Health Plan 2004–2010 [22, 66] represented a second planning effort. It raised the issue of skill mix and new competencies needs, but did not go “much beyond a call for a more explicit strategy” [11]. In 2012, the National Health Plan 2012–2016 [67], following recommendations of the World Health Organization [25, 42], included the objective of designing and implementing a HRH policy [23]. However, the targets set in the Plan were restricted to numbers of physicians per inhabitant, from 3.83 physicians per 1000 habitants in 2009 to 4.51 in 2016 [23, 68].

To date, no HRH policy has been formulated [23, 25, 42], in spite of numerous policy documents and reports stating that it is needed. On the other hand, various isolated and ad hoc strategies have been adopted.

Strategies to address shortage included an increase in the numerus clausus, the opening of new medical schools and programmes, an increase of residency places and the re-hiring physicians who had retired from the NHS. This last strategy was also intended to reduce the level of care maldistribution of physicians.

Since 1999, there has been a gradual rise in the numerus clausus; in 2010, it was 2.5 times higher than 15 years before [69], whereas population growth during that period was 3.5% [47]. Until 1999, Portugal had five medical schools, two in Lisbon, two in Oporto and one in Coimbra offering a total of 566 places [14, 70]. Two new medical programmes were opened in 2001 in universities of the interior of the country [9, 14]. Additionally, a course exclusively for students holding a bachelor´s degree opened in 2009 in Algarve to attract young professionals to the south of the country [70]. In 2004/2005, basic courses were created in Azores and Madeira Islands; students complete the first 3 years of medical education there and the remaining years at the University of Coimbra [10, 70].

There was also an increase in the number of residency slots for specialization, after the compulsory post-graduation of general internship that includes a total of 6 months involving general practice and public health and a year of hospital-based training. It is followed by a period of 4 to 6 years of training for a medical specialty [14, 37, 69]. There are three medical career streams: hospital-based practice (45 specialties), public health and general practice [10, 71]. The MoH defines the number of residency places in consultation with the Medical Council, depending on the available training capacity in recognized provider organizations [40, 69]. The number of residency places grew from 894 in 2006 [69] to 1569 in 2015 [72].

The re-hiring of retirees for a period of 3 years was authorized in June 2010 [35]. It aimed to overcome the shortage of physicians [37, 73, 74], particularly of GPs [35, 73]. In 2013, this strategy was extended for another 3 years [21, 74]. It is applicable to any retired physician, including those who anticipated their retirement. The benefits include the accumulation of the pension with a third of the remuneration according to contracted hours [73].

We identified four strategies addressing the geographical maldistribution of physicians: reserved vacancies, the “partial mobility of professionals”, financial and non-financial incentives during a 5-year period to work in an underserved area and four bilateral agreements to recruit physicians from other countries to work in PC in underserved areas.

In 1975, a strategy referred to as the “Medical Service in Peripheries” introduced a 1-year compulsory service outside urban areas [75, 76]. This was implemented until 1982. In 2009, financial incentives were introduced for resident physicians who commit to work in an underserved area or specialty after graduation during a period equal to their specialist medical training programme; this policy was referred to as “reserved vacancies” [40, 77, 78]. It consisted in a monthly residency grant of €750, paid by the municipality where the physician committed to work [79]. In case of failure to fulfil the obligation, the resident had to repay the grant [77]. No evaluation of this measure has been identified. This strategy is now restricted to the Azores and Madeira Islands.

The “partial mobility of physicians” regulation, approved in 1998 and updated in 2015 [80], is a special arrangement that provides for a daily allowance and transport subsidy (€200 per day) accessible to physicians who work part-time in two or more public services more than 60 km apart; it is particularly used in the region of Algarve, which experiences important seasonal variations in its resident population [73, 80].

In 2015, a new law created a set of financial and non-financial incentives to attract and retain physicians in poor and underserved areas [20]. These incentives target physicians working in a NHS establishment in an underserved area [20]. Under this scheme, physicians who accept a 5-year contract receive an additional €1000 for the first 6 months, then €500 for the next 6 months and €250 per month for the remaining 4 years [20]. Additional incentives are available, such as child’s school transfer guarantee, support to spouse employment and an extra 2 days of annual leave [20, 73]. Penalties and reimbursement are imposed in case of non-compliance [20].

Since 2008, the MoH has used an “emergency measure” in the form of the recruitment of foreign physicians through bilateral agreements with Latin American countries [10, 23, 25, 57, 81]. This option was chosen because attracting workers from other European countries proved difficult as Portugal could not offer conditions competing with those offered by countries like Germany or England [82]. The first bilateral agreement was signed with the government of Uruguay in 2008; 15 physicians came to work for the National Institute of Medical Emergencies [83–85]. In 2009, another one was signed with Cuba, and 44 physicians arrived to work in five health centres in Algarve, nine in Alentejo and one in LVT [86–88]. They returned to Cuba in 2012 and were replaced by another cohort [89, 90]. Another replacement took place in 2014 [91–93]. In 2011, bilateral agreements with Colombia and Costa Rica brought 82 and nine physicians, respectively, to health centres in the LVT and Central regions [85, 94–97]. Foreign medical degrees were validated by the Faculty of Medicine of Porto, and registration with the Medical Council followed [83, 95, 98, 99]. Prior to arrival, the physicians attended a Portuguese language course [83, 95, 100]. They also had a 2-week period for adaptation/integration into the services [95]. There has been no evaluation of the efficiency or effectiveness of these recruitments. A study that assessed the foreign physicians’ cultural competencies concluded that these health professionals performed in a culturally competent manner and contributed positively to improving access to health services [81].

Four strategies aimed at changing the distribution of physicians by level of care. First, the PC reform was designed as a strategy to increase the recognition of GPs’ career and to improve accessibility to primary level services. Second, in 2007, a quota of 25% of residency places for GPs was established [37, 40, 101]. Over the years, this has contributed to augmenting the number of GPs, but it has not been sufficient to extend PC coverage to the whole population [54, 102, 103]. In 2015, 12% of the population was still without access to a GP, ranging from 3.3% in the North to 25.8% in Algarve [104]. Third, the bilateral agreements to recruit foreign physicians aimed at addressing, at the same time, geographical and level of care maldistribution as they concerned only family practitioners, and finally, there was the re-hiring of retired physicians which focused on family physicians. The interventions to address supply, geographic and level of care maldistribution by area of political intervention are presented in Additional file 3: Table S2.

## Discussion

In Portugal, like in any country with a national health system offering universal coverage, policy and decision-makers are challenged to ensure the availability and accessibility of physicians in all geographical areas, at the most appropriate level of care.

The “shortage” of physicians is not related to their insufficient total number, as shown by ratios to population above the EU average, but to the unwillingness of these professionals to work in certain zones of the country. The lack of physicians at the PC level is better explained by the insufficient number of residency places and the age structure of GPs than by the unavailability of physicians. Even though this has been documented and highlighted by many observers, there is still a lack of evidence-informed policies to address these problems.

Workforce policies limited to training more physicians are not sufficient to address shortages as they ignore the dynamics of health labour market [28]. For instance, whether market conditions are adequate or not to attract and absorb the newly qualified professionals is critical to the decisions by future physicians as to which specialty to choose, where to work and even to stay in the country [28]. The absence of data on future graduates’ expectations and intentions as to their professional life makes it difficult to adjust policies of attraction and retention to ensure that population needs will be met. Without a valid diagnosis of the current situation and a comprehensive and up-to-date database, policies risk to be developed in an improvised rather than in an informed manner.

The implementation by the Portuguese government of the various health workforce interventions mentioned here has generated debate. Medical organizations “have unsuccessfully opposed” those targeting geographical imbalances using the argument that the lack of incentives for physicians to work in underserved areas was the main problem, not their scarcity [12]. There is a paradox in having difficulty in recruiting physicians in some regions and in PC and having hundreds of Portuguese physicians migrating to other European countries as well as to Australia, the Gulf States and even Brazil [23, 40]. There is also a paradox in recruiting from Latin America, while hundreds of young Portuguese study medicine in countries such as Hungary, Slovakia, France, Spain and the Czech Republic and return to Portugal at the time of specialization [41]. Further analysis is needed of the factors that influence the choice of physicians to migrate as well as of those which bring them to avoid work in underserved areas. Qualitative studies and discrete choice experiments should be prioritized as they help identify and understand the preferences of future professionals and of those already in practice [105, 106].

The same reasoning applies to how to address the imbalanced distribution between PC and hospital services. The reasons for this maldistribution are considered to be the low prestige of PC, the lack of planning and coordination of training and the limited number of internship places in family medicine. Only the last one was addressed, in a partial manner, by raising the quota of residency places in family medicine. The effects of this measure and the bilateral agreements to recruit abroad and the re-hiring of retired GPs have yet to be properly assessed.

Interventions to tackle the challenges reviewed here have been implemented in an isolated manner. Evidence on the subject indicates that combinations of actions are more effective than isolated ones [4]. Also, all these interventions focused on the provision of services by the public sector, whereas the private sector occupies an important space in the health labour market in Portugal and influences physicians’ career choices.

There is a diversity of theories and analytical frameworks seeking to explain a policy process. The most common approach disaggregates the process into a number of functional steps [107]. An example is the policy cycle, which is typically divided into four stages: agenda setting, formulation policy development, implementation and evaluation [108–110]. The policy process is influenced by different factors [111, 112], and this is where scientific evidence can play a significant role. At each stage of the policy cycle, there is a corresponding stage of research that can serve to inform the policy process [113, 114]. For example, when a problem is not on the agenda and needs to be brought to the attention of policy-makers, research that documents it and identifies its causes and the need for action is useful. Research can also contribute to identifying policy options and their relative capacity to address the problem as well as the risks involved in their implementation.

Furthermost, research on health workforce in general and on physicians in particular is limited in Portugal. Demand-on the part of policy and decision-makers- is weak as illustrated by the low priority which the main source of financial support for research in Portugal, the Foundation for Science and Technology, gives to health service research, let alone to research on health workforce issues [23].

This study based on documentary analysis has limitations because we cannot pretend to have a comprehensive perspective of the Portuguese health policy context only on the basis of written sources. This is why an additional phase of our research, consisting in interviews with key policy and decision-makers and researchers, is in development.

## Conclusions

In this paper, we reported that policy and decision-makers face challenges to assure geographical access to physicians in the Portuguese NHS, because of geographical and level of care imbalances, and that strategies to tackle these challenges are sparse and not evaluated. Furthermore, research in human resources and distribution of physicians, in particular, is limited in Portugal.

More investment is therefore needed in research to analyse the causes of maldistribution in Portugal, particularly on individual and professional factors that influence the choice of a location of practice and on interventions to mitigate the problem. This would provide the basis for comprehensive evidence-based health workforce policy development. Also, it is necessary to have tools in place to help identify, analyse and evaluate research conducted in other contexts and adapt its results to the Portuguese context and needs.

## Additional files

Additional file 1: Table S1. Search strategy [47, 104, 115–122]. (DOCX 18 kb)

Additional file 2: Figure S2. Flowchart of peer-reviewed articles search results (BVS and PubMed). (JPG 145 kb)

Additional file 3: Table S2. Interventions to address supply, geographic and level of care maldistribution by area of political intervention [2, 5, 123]. (DOCX 18 kb)
