# Supplementary material for: Challenges and strategies to improve the availability and geographic accessibility of physicians in Portugal
Source: Hum Resour Health. 2017 Mar 23;15:24. doi: 10.1186/s12960-017-0194-3 (PMC5364681; doi:10.1186/s12960-017-0194-3)
Supplement: Supplementary file 1 — Search strategy [47, 104, 115–122]. (DOCX 18 kb) [file 12960_2017_194_MOESM1_ESM.docx]

Additional file 1: Table S1. Search strategy

**Table S1.** Search strategy

| **Sources of information** | **Search terms** | **Inclusion criteria** | **Exclusion criteria** |
| --- | --- | --- | --- |
| PubMed and BVS | The terms used to search online databases were: (physician OR doctor OR "health professional" OR “workforce” OR médico OR "profissional de saúde" OR "profissional da saúde") AND Portugal. | (i) document must be in the public domain,  (ii) available in an electronic format,  (iii) published after 1995*,  (iv) political documents addressing health workforce issues,  (v) article addressing the availability or geographic accessibility of health workers,  (vi) documents should address the health workforce situation in Portugal separately from other countries. | (i) document not in the public domain,  (ii) not available in an electronic format,  (iii) published before 1995,  (iv) documents not addressing health workforce issues,  (vi) documents not including information on Portugal, |
| Websites | Government websites - Ministry of Health [115,116], The Directorate-General of Health [117], National Health Plan 2012-2016 [118] and Central Administration of the Health System (*Admistração Central do Sistema de Saúde* – ACSS) [104]; Medical Council [119], Portuguese Health System Observatory (*Observatório Português dos Sistema de Saúde - OPSS)* [120], PORDATA (Fundação Francisco Manuel dos Santos) [47]. Additional websites were accessed for specific country information – World Health Organization (WHO) [121] and Organization for Economic Co-operation and Development (OECD) [122]. |  | (vii) documents reporting data without any analysis. |
| Online newspapers | Público, Correio da Manhã and Jornal de Notícias. Key-words were: médicos uruguaios, médicos cubanos, médicos colombianos, médicos costa-riquenhos, médicos porto-riquenhos, médicos chilenos, acordos bilaterais médicos and homologação de título(s) médico(s). |  |  |

*Documents dated before 1995 and deemed relevant for the understanding of interventions adopted later were also considered.
