# Supplementary material for: Challenges and strategies to improve the availability and geographic accessibility of physicians in Portugal
Source: Hum Resour Health. 2017 Mar 23;15:24. doi: 10.1186/s12960-017-0194-3 (PMC5364681; doi:10.1186/s12960-017-0194-3)
Supplement: Supplementary file 3 — Interventions to address supply, geographic and level of care maldistribution by area of political intervention [2, 5, 123]. (DOCX 18 kb) [file 12960_2017_194_MOESM3_ESM.docx]

Additional file 3

**Table S2.** Interventions to address supply, geographic and level of care maldistribution by area of political intervention

|  | Challenges | | | | Areas of Political Interventions | | | |
| --- | --- | --- | --- | --- | --- | --- | --- | --- |
| Political Intervention | Geographical distribution | Level distribution | Shortages of physicians | Policies on production | | Address inflows and outflows | Address maldistribution and inefficiencies | Regulate the private sector |
| a) Increase in the numerus clausus - 1998 |  |  | ✓ | ✓ | |  |  |  |
| b) Opening of new medical schools - 2001 and 2004 |  |  | ✓ | ✓ | |  |  |  |
| c) Increase of residency places - 2006 |  |  | ✓ | ✓ | |  |  |  |
| d) Regulation of re-hiring of retired physicians (benefits include accumulation of the pension with a third of the remuneration) - 2010 |  | ✓ | ✓ |  | | ✓ |  |  |
| e) Reserved vacancies (receive a monthly amount of residency grant) - 2004 | ✓ | ✓ |  | ✓ | |  | financial* |  |
| f) Partial Mobility of professionals (sets an amount of daily allowance and applicable transports) - 1998 | ✓ |  |  |  | |  | financial* |  |
| h) Financial and non-financial incentives to work in an underserved area (non-financial incentives, such as children’s school transfer guarantee and increasing the holiday leave by two days) - 2015 | ✓ |  |  |  | |  | financial and personal and professional support* |  |
| h) Bilateral agreement Uruguay - 2008 | ✓ | ✓ |  |  | | ✓ | bilateral agreement* |  |
| h) Bilateral agreement Cuba -2009, 2012 e 2014 | ✓ | ✓ |  |  | | ✓ | bilateral agreement* |  |
| h) Bilateral agreement Colombia - 2011 | ✓ | ✓ |  |  | | ✓ | bilateral agreement* |  |
| h) Bilateral agreement Costa Rica - 2011 | ✓ | ✓ |  |  | | ✓ | bilateral agreement* |  |
| j) Primary health care reform - 2005 |  | ✓ |  |  | |  | personal and professional support* |  |
| i) Quota of 25% of residency places for GPs - 2006 |  | ✓ |  | ✓ | |  | administrative* |  |

*Interventions (policy and regulation) to attract, recruit or retain the health workers to a determinant region, level or specialty can be divided into five categories specifically to tackle the geographical workforce maldistribution. The areas of action of interventions are: regulatory administrative; educational; financial; personal and professional support [2,5,123].
